# Supplementary material for: Reduced excitatory neuron activity and interneuron-type-specific deficits in a mouse model of Alzheimer’s disease
Source: Commun Biol. 2022 Dec 2;5:1323. doi: 10.1038/s42003-022-04268-x (PMC9718858; doi:10.1038/s42003-022-04268-x)
Supplement: Supplementary file 2 — Supplementary Information [file 42003_2022_4268_MOESM2_ESM.pdf]

Supplementary Information
for
Reduced excitatory neuron activity and interneuron-type-specific deficits in a mouse model of Alzheimer's disease

Moustafa Algamal[1], Alyssa N. Russ[1], Morgan R. Miller[1], Steven S. Hou[1], Megi Maci[1], Leon P. Munting[1], Qiuchen Zhao[1], Dmitry Gerashchenko[2], Brian J. Bacskai[1]*, Ksenia V. Kastanenka[1]*

[1]Department of Neurology, MassGeneral Institute of Neurodegenerative Diseases, Massachusetts General Hospital and Harvard Medical School, Charlestown, MA, USA.

[2]Harvard Medical School/VA Boston Healthcare System, West Roxbury, MA, USA.

*Corresponding authors: KKASTANENKA@mgh.harvard.edu, BBACSKAI@mgh.harvard.edu

Supplementary Figures

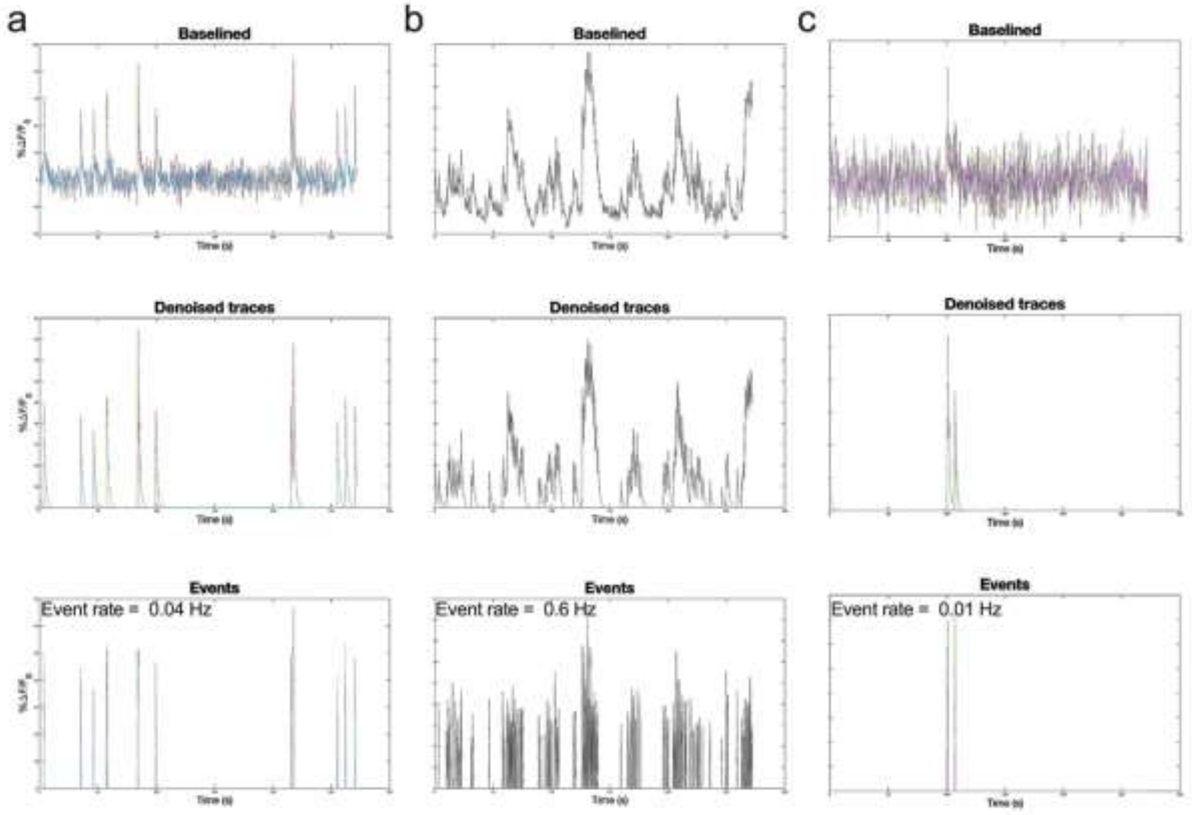

Supplementary Figure 1. Examples of deconvoluted calcium events in SOM interneurons. Top, Baselined raw calcium traces from three SOM cells (**a**, **b** and **c**). See also a recording of the same cells in Movie 1. Middle, Denoised calcium traces from the same cells depicted in the top panels. Bottom, estimation of deconvolved calcium event rates after thresholding spikes to two and half times the standard deviation of the noise. We ran OASIS using the following MATLAB implementation: [c_oasis, s_oasis, options] = deconvolveCa(y, 'ar1', g, 'foopsi', 'lambda', lambda, 'smin', smin). Lambda, the sparsity regularization parameter, was set to 0 while smin was set to -2.5.

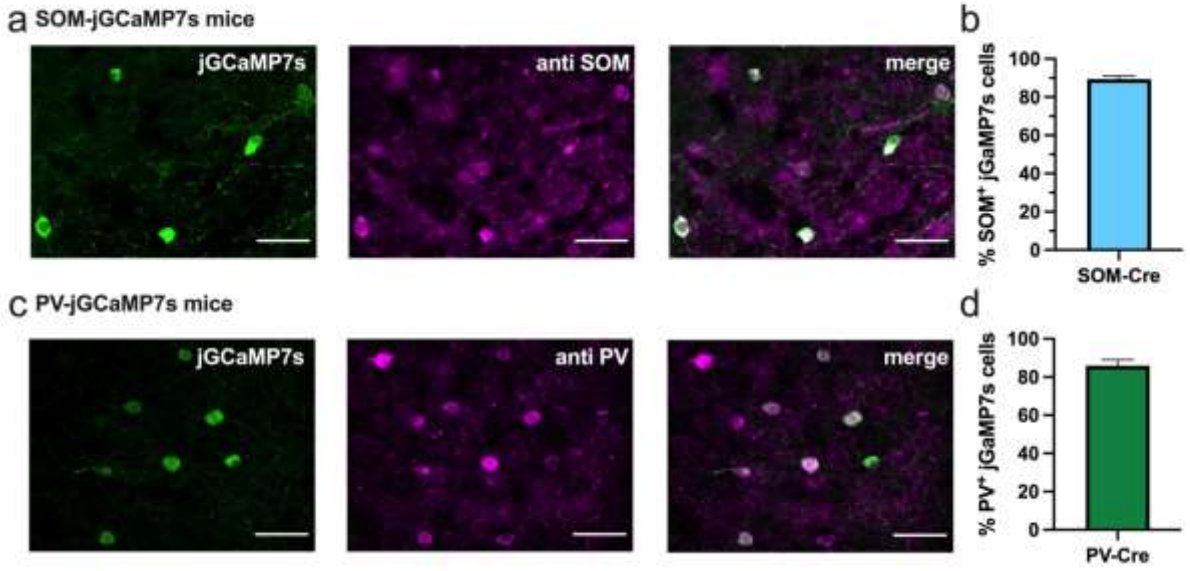

Supplementary Figure 2. Expression of jGCaMP7s in PV-Cre and SOM-Cre mice. Confocal images of a brain slice from a SOM-Cre mouse injected with FLEX-jGCaMP7s showing green jGCaMP7s (anti-GFP staining), SOM (anti-SOM staining) in magenta, and the merged image (**a**). Quantification of SOM positive cells show that 90 ± 1.6% of jGCaMP7s cells are SOM positive in SOM-cre mice (**b**). Confocal images of a brain slice from a PV-Cre mouse injected with FLEX-jGCaMP7s showing green jGCaMP7s (anti-GFP staining), PV (anti-PV staining) in magenta, and the merged image (**c**). Quantification of PV positive cells show that 86 ± 3.2% of jGCaMP7s cells are PV positive in PV-Cre mice (**d**). All error bars reflect the mean ± s.e.m.

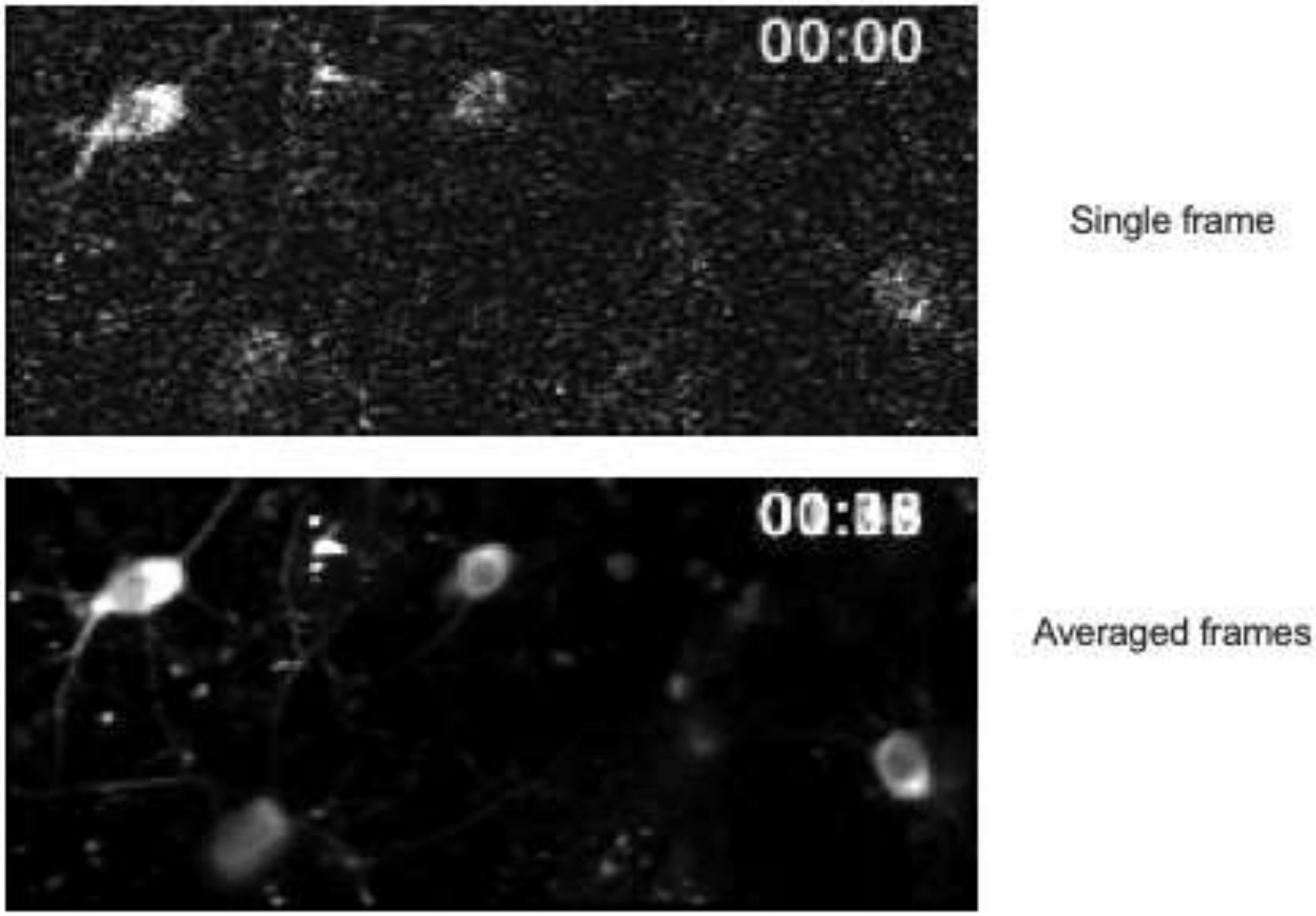

Supplementary Figure 3. Upper, single frame image of PV cells from Movie2 at 256x256 resolution. Lower, an image crated after averaging 500 frames to improve signal to noise.

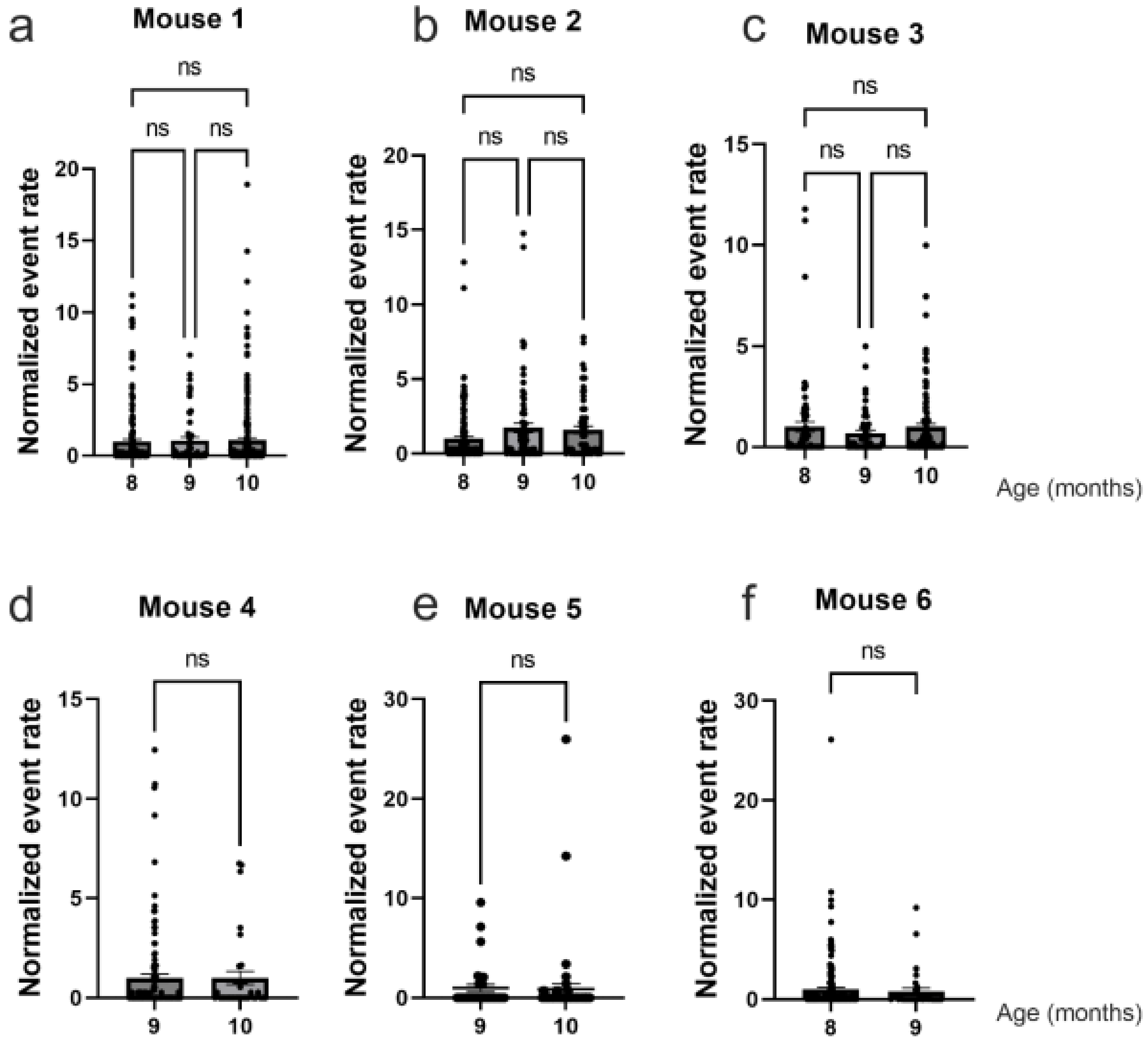

Supplementary Figure 4. Stability of neuronal event rates with age. We monitored the activity of excitatory neurons in APP mice over multiple imaging sessions to examine the impact of aging on mean Ca+2 event rates. All event rates were normalized to the mean event rate during the first imaging session. No significant changes were observed in event rates in APP mice receiving multiple imaging sessions at 8, 9, or 10 months of age. Panels a-c were analyzed using One-way ANOVA followed by Dun's test to correct for multiple comparisons, while panels e-f were analyzed using a t-test. Each bar represents the mean event rates per mouse, and all error bars reflect the mean ± s.e.m. 'ns' denotes no significance p>0.05.

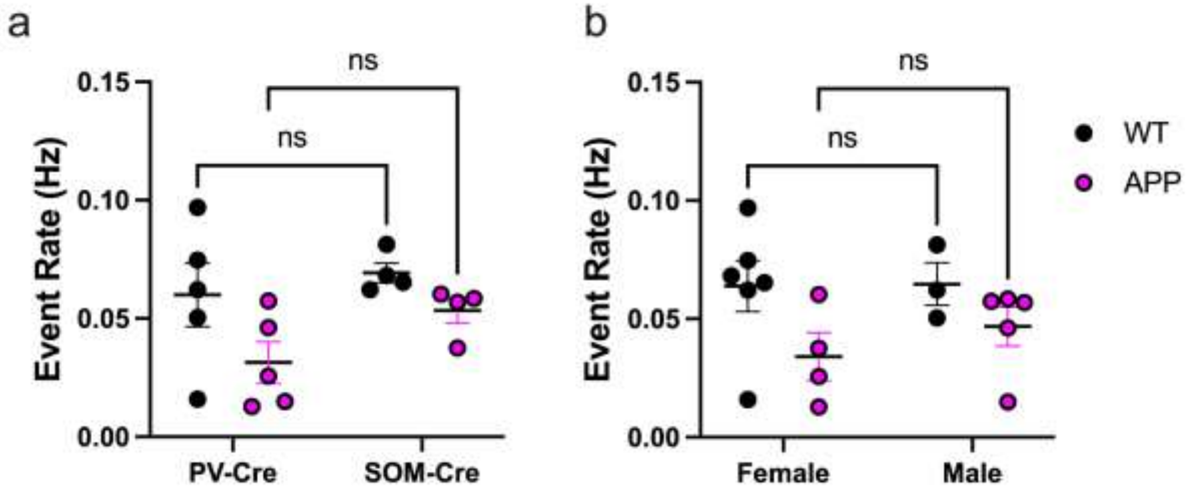

Supplementary Figure 5. Effect of mouse model and sex on excitatory cell event rates. a, Mean event rates for excitatory cells from PV-Cre and SOM-Cre WT and APP mice (Two-Way ANOVA, main PV-Cre vs. SOM-Cre effect $F_{(1, 14)} = 0.4$, $p = 0.54$, main WT vs. APP effect $F_{(1, 14)} = 5.07$, $p = 0.041$, main interaction $F_{(1, 14)} = 0.31$, $p = 0.58$). b, Mean event rates for excitatory cells in male and female WT and APP mice (Two-Way ANOVA, main male vs. female effect $F_{(1, 14)} = 2.6$, $p = 0.18$, main WT vs. APP effect $F_{(1, 14)} = 5.4$, $p = 0.035$, main interaction $F_{(1, 14)} = 0.44$, $p = 0.52$). All error bars reflect the mean ± s.e.m.

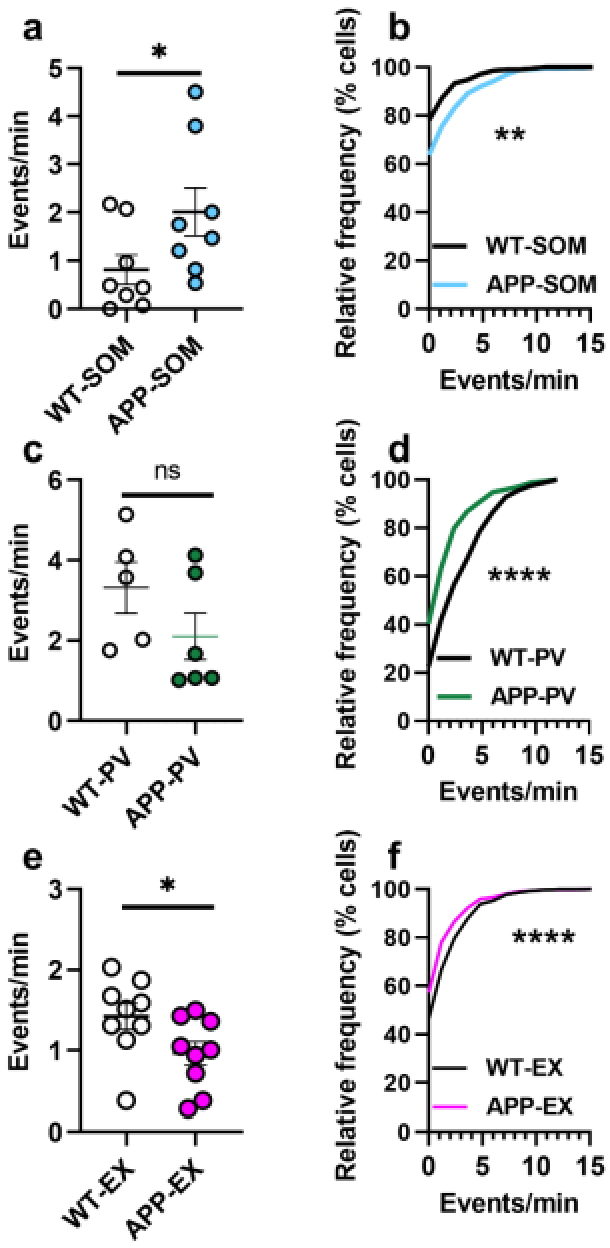

Supplementary Figure 6. Cell-type-specific calcium event rates using peak counting. The data presented in Figures 1-3 of the original manuscript were reanalyzed using the method described by Korzhova et al. 2021. In brief, after estimating baseline noise and smoothing over five frames, peaks with intensities 3x higher than noise standard deviation and a minimum distance of 1.5 seconds were counted. a, Mean neuronal calcium event rates in SOM interneurons (n= 8 WT-SOM mice; 8 APP-SOM mice). b, Cumulative frequency distribution of calcium event rates in all imaged neurons (n= 210 WT-SOM neurons; 364 APP-SOM neurons). c, Mean neuronal calcium event rates in PV interneurons (n= 5 WT-PV mice; 6 APP-PV mice). d, Cumulative frequency distribution of calcium event rates in all imaged neurons (n= 254 WT-PV neurons; 172 APP-PV neurons). e, Mean neuronal calcium event rates in SOM interneurons (n= 9 WT-EX mice; 9 APP-EX mice). **f**, Cumulative frequency distribution of calcium event rates in all imaged neurons (n= 1412 WT-EX neurons; 1603 neuron APP-EX neurons).

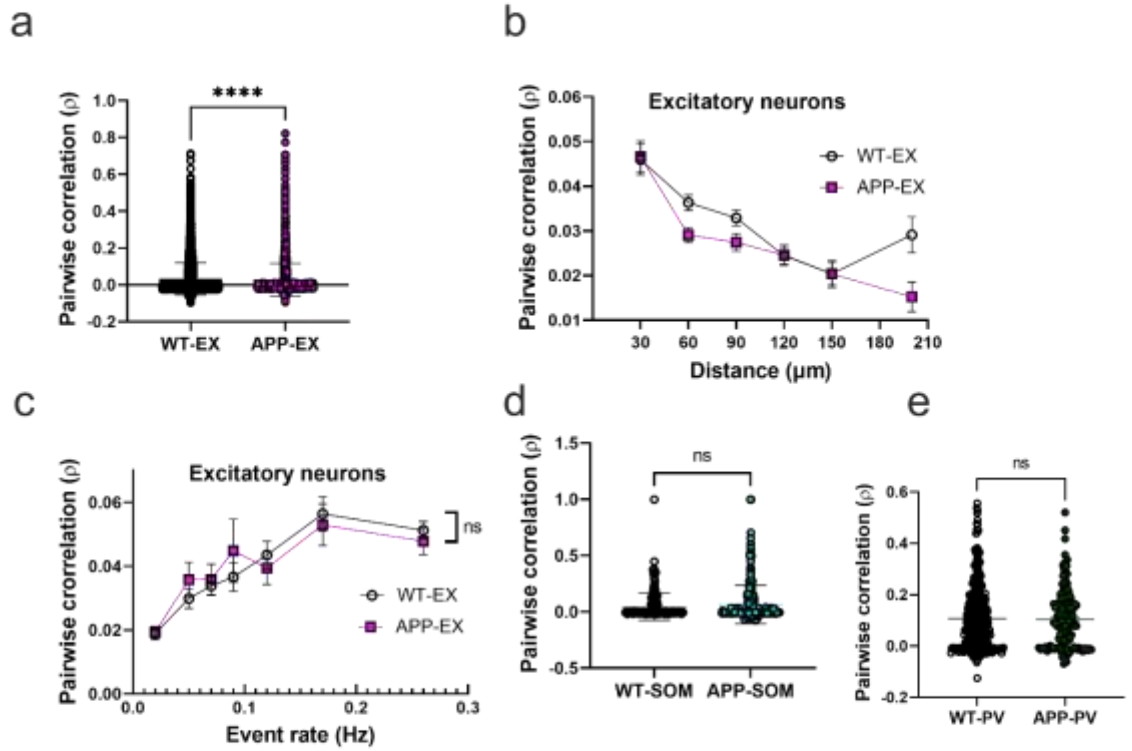

Supplementary Figure 7. Pairwise neuronal correlations in APP/PS1 mice. **a,** Average pairwise Pearson correlation values for excitatory neurons in APP/PS1 and WT mice (Mann–Whitney *U* = 3012, n = 8791 WT-EX pairwise-correlations, n = 8409 APP-EX pairwise- correlations, *p*<0.0001, two-tailed). **b**, Relationship between pooled pairwise Pearson correlation values and pairwise interneuronal distance (Two-Way ANOVA, main distance effect $F_{(5, 17193)}$ = 21.78, p = <0.0001, main genotype effect $F_{(1, 17193)}$ = 7.066, p = 0.008, main genotype and distance interaction $F_{(5, 17193)}$ = 1.72, p = 0.128). The x axis values represent the maximum limit of the pooled pairwise distances. **c**, Relationship between correlation values and event rates after pooling data according to geometric means of neuronal event rates (Two-Way ANOVA, main event rate effect $F_{(6,2211)}$ = 29.07, p = <0.0001, main genotype effect $F_{(1, 2211)}$ = 0.74, p = 0.427, main genotype and event rate interaction $F_{(6, 2211)}$ =0.55, p =0.77). **d-e**, Average pairwise Pearson correlation values for SOM interneurons (**d**) (Mann–Whitney *U* = 254487, n = 163 WT-SOM pairwise-correlations, n = 337 APP-SOM pairwise-correlations, *p*= 0.193, two-tailed) and PV interneurons (**e**) (Mann–Whitney *U* = 32424, n = 392 WT-PV pairwise-correlations, n = 166 APP- PV pairwise-correlations, *p*= 0.95, two-tailed) in APP/PS1 and WT mice. All error bars reflect the mean ± SD.

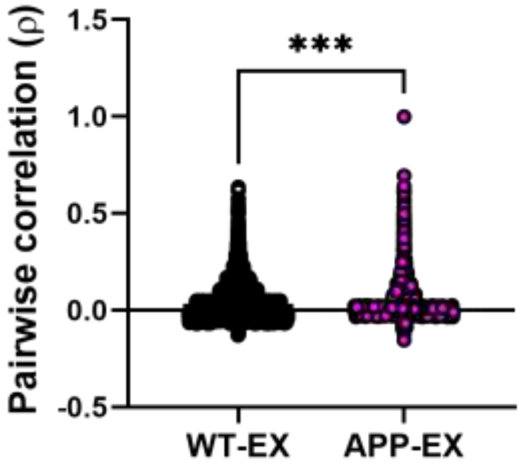

Supplementary Figure 8. Average pairwise correlations for excitatory neurons after eliminating all pairwise correlations form neurons at distances longer than 150 μm.
