# Supplementary material for: Reduced excitatory neuron activity and interneuron-type-specific deficits in a mouse model of Alzheimer’s disease
Source: Commun Biol. 2022 Dec 2;5:1323. doi: 10.1038/s42003-022-04268-x (PMC9718858; doi:10.1038/s42003-022-04268-x)
Supplement: Supplementary file 3 — Description of Additional Supplementary Files [file 42003_2022_4268_MOESM3_ESM.pdf]

Description of Additional Supplementary Files

File name: Supplementary Movie 1

Description: Spontaneous activity of jgcamp7s-expressing somatostatin interneurons in an APP/PS1 mouse under 1% isoflurane anesthesia.

File name: Supplementary Movie 2

Description: Spontaneous activity of jgcamp7s-expressing parvalbumin interneurons in a non-transgenic mouse under 1% isoflurane anesthesia.

File name: Supplementary Movie 3

Description: Spontaneous activity of gcamp6s-expressing pyramidal neurons in a non-transgenic mouse under 1% isoflurane anesthesia.

File name: Supplementary Movie 4

Description: Spontaneous activity of gcamp6s-expressing pyramidal neurons in an APP/PS1 mouse under 1% isoflurane anesthesia. Amyloid plaque is shown in magenta.
